# Big Data in Context: Addressing the Twin Perils of Data Absenteeism and Chauvinism in the Context of Health Disparities Research

**DOI:** 10.2196/16377

**Published:** 2020-01-07

**Authors:** Edmund W J Lee, Kasisomayajula Viswanath

**Affiliations:** 1 Dana-Farber Cancer Institute Boston, MA United States; 2 Harvard TH Chan School of Public Health Boston, MA United States; 3 Wee Kim Wee School of Communication and Information, Nanyang Technological University Singapore

**Keywords:** big data, artificial intelligence, health informatics, wearable electronic devices, mobile health, social media, electronic health records, digital divide, health disparities

## Abstract

Recent advances in the collection and processing of health data from multiple sources at scale—known as big data—have become appealing across public health domains. However, present discussions often do not thoroughly consider the implications of big data or health informatics in the context of continuing health disparities. The 2 key objectives of this paper were as follows: first, it introduced 2 main problems of health big data in the context of health disparities—data absenteeism (lack of representation from underprivileged groups) and data chauvinism (faith in the size of data without considerations for quality and contexts). Second, this paper suggested that health organizations should strive to go beyond the current fad and seek to understand and coordinate efforts across the surrounding societal-, organizational-, individual-, and data-level contexts in a realistic manner to leverage big data to address health disparities.

## Introduction

The emergence of big data platforms is showing promise in addressing many public health problems, such as predicting and managing the spread of global infectious diseases by drawing on real-time data on social media [1,2], empowering people to monitor their health through wearable technologies and interact with health care providers through patient portals [3,4]. Big data are defined as extensive datasets characterized by 5 *Vs*—v*olume* (size of the data), *velocity* (speed at which data are collected and processed), *variety* (types of data), *veracity* (trustworthiness of the data), and *value* (usefulness for decision making), which would require sophisticated computing infrastructure for storage, management, and analysis [5,6]*.* Although many are optimistic about big data in bringing significant improvements to individuals’ health [7,8], others have argued that implementation of big data or health informatics interventions could increase health inequality [9].

This is because health organizations (eg, hospitals, nongovernmental organizations, federal public health agencies, and academic institutions) that want to incorporate the use of big data in their work with underprivileged groups may arguably face additional challenges beyond computational complexities. In addition to the day-to-day data wrangling and predictive optimization, organizations working in public health also need to deal with the challenges, such as difficulty in recruiting, retaining, and obtaining data from population groups that have suffered disproportionately from disease burden. It is thus important to consider what are the challenges that impede underprivileged groups in achieving equitable health outcomes. This is critical to the success of deploying big data solutions to improve the health of underprivileged groups, as they may not have the resources to access some of the communication technologies (eg, wearable gadgets and smartphones) that are used as apparatus for health data collection, leading to a new digital data divide [10].

The objectives of this commentary were 2-fold. First, we have presented 2 emerging challenges—*data absenteeism* and *data chauvinism*—that could significantly dilute the effectiveness of big data and health informatics initiatives in health disparities research. Second, we have argued that organizations involved in public health work should strive to understand the collection and use of big data in different contexts and coordinate efforts across societal, organizational, individual, and data levels to effectively address health disparities.

## The Perils of Big Data: Absenteeism and Chauvinism

Any attempts to draw on big data to address health disparities will face enormous challenges. Referring to underprivileged groups, a recent report by the United States Agency for International Development in identifying challenges to big data implementation in resource-poor settings underscored 2 main obstacles: the quantity and quality of data from the poor [11]. From the get-go, data from the poor are often not represented because of the lack of cyberinfrastructure in some locations or the poor might not have access to the technologies required for their data to be captured. Even if the poor are represented, the data are often messy and incomplete, and blind faith in these data points—even if they are voluminous—would lead to biased results and inaccurate interpretations [12]. These problems related to the quantity and quality of data are characterized as *data absenteeism* and *chauvinism*, respectively.

Data absenteeism describes an ironic phenomenon of data scarcity in a data-rich society, where data from underprivileged groups are not represented—or severely underrepresented—in the databases of health organizations [13]. For instance, a study on the diversity and representation of racial groups across 51 biobanks in the United States found that compared with the US census, there were statistically significantly lower enrollment numbers for Hispanics and Latinos (US census: 18%; selected biobanks: 7%), as well as Hawaiian and Pacific Islanders (US census: 0.2%; selected biobanks: 0.01%) [14]. In another example, a study using the Healthcare Effectiveness Data and Information Set (2012-2015) found that in the United States, less than half of Medicare, Medicaid, and commercial insurance plans reported complete or partially complete data on ethnicity of their members, although the Affordable Care Act specifically required population health surveys in federal health programs to collect and report items on race, ethnicity, and language as part of the drive to reduce health disparities [15]. In addition, research has shown that the users of health technologies (eg, mobile health apps) are more likely to be younger, be highly educated, and have higher levels of digital literacy skills [16], and it is not known how represented underprivileged groups are in health interventions using health technologies.

This is a major issue that has been repeatedly documented as underprivileged groups may be overlooked or may not have easy access to big data platforms or devices that are often used for collecting data [17], be it social media [18], smartphones [16], or internet patient portals [4]. Even if accessibility is not a problem, the underprivileged groups would still face additional barriers. For instance, a study on the use of internet patient portals by a population of diabetic adults in Northern California showed that racial minorities were more likely to request for password reset when accessing internet patient portals and logged on less, suggesting that even with access, they were still left behind [19].

Drawing from the ecological perspective of health, there is a myriad of societal, organizational, and individual factors that collectively explain why data from underprivileged groups are not represented [20]. On a societal level, *social determinants* (eg, education level and economic and employment status) and *communication inequalities*—unequal access to and use of communication technologies—are contributing factors to data absenteeism. For instance, underprivileged groups may struggle with having access to necessities and infrastructure, such as sanitation, water, and proper housing, and having the latest digital communication devices may not rank high compared with these basic necessities. In addition, many of these digital technologies (eg, wearable gadgets) used for collecting public health big data may simply be out of reach for groups from lower socioeconomic position because of the cost factor. From an organizational perspective, using big data to address health disparities may be perceived as a costly long-term investment, and many small health organizations at the community level that cater to underprivileged groups do not have the *capacity* (eg, comprehensive data architecture) or the human *capital* (eg, staff who know how best to turn data into insights to benefit the organization operationally) to do so. On the individual level, recent high-profile scandals on misuse of data on social media, such as the Facebook-Cambridge Analytica scandal [21], could further erode trust in health systems.

Data absenteeism has serious ramifications, and it has a profound impact on underprivileged groups even if the effects are not visible or tangible in the short run. As government and public health systems are increasingly using big data to automate solutions for decisions pertaining to who would get public assistance and financial aid, data absenteeism could further penalize the underprivileged groups. These groups that require the most financial assistance for health and medical services would not be in the very system to contribute to the development of the machine learning algorithms to identify them and further deprive them of the assistance they need.

## The Perils of Data Chauvinism

The second peril that threatens big data’s efficacy in addressing health disparities is the problem of data chauvinism. Data chauvinism is the overconfidence that the acquisition of (big) data alone would be the panacea to health disparities, without due consideration for ensuring data quality when collecting data from the underprivileged groups. Clearly, the weaknesses and cracks of data chauvinism are visible in the light of some of the failings of high-profile projects, such as the Google Flu Trend (GFT) study, which overestimated the prevalence of flu compared with the Centers for Disease Control and Prevention’s (CDC) estimates from traditional reports from laboratories [22]. Certainly, quantity is not synonymous with quality, as fundamental threats to validity and reliability, such as data noise, confounds, and spurious relationships, needed to be accounted for when designing big data research and solutions. In the context of health disparities, the efficacy of big data in building models to predict outcomes may come under threat because of biases, such as self-selection and the lack of generalizability, resulting in overfitting of data [23].

As machine learning algorithms are typically trained on a training set before being applied to the test set, if there are inherent biases—reflected in the absence or incomplete data—in the training set, it would severely compromise the quality and accuracy of the prediction outcomes. Such instances of quantified discrimination have real-world repercussions and may further punish the underprivileged groups. In Indiana’s experiment with welfare eligibility automation, some from the underprivileged groups lost their Medicaid benefits because the algorithms wrongly diagnosed them as *failing to cooperate*, thus disqualifying them from receiving the benefits [24].

## Toward Understanding Big Data in Context

Recognizing the twin perils of data absenteeism and data chauvinism in the context of big data use for health disparities research, what steps could organizations take to address them considering that many are moving toward the integration of big data solutions into their system? There are no obvious and easy solutions, but we suggest that health organizations should strive to go beyond solely cultivating computational competency and consider societal-, organizational-, individual, and data-level contexts when implementing big data research and solutions to address health disparities to avoid the pitfalls of data absenteeism and data chauvinism.

## Societal-Level Context: Addressing Social Determinants and Communication Inequalities

First, to combat data absenteeism and data chauvinism when designing big data research or health informatics interventions, health organizations should seek to understand how societal-level contexts, such as social determinants and communication inequalities, are barriers to the underprivileged in reaping the benefits of big data. In the context of interventions using smartphones or wearable gadgets, researchers need to be mindful that providing access to digital devices does not fully remove structural obstacles for the underprivileged groups. Apart from the costs of purchasing digital devices, the poor would need to bear additional recurring costs that are often minute from the perspective of the average working class. These are known as *connection maintenance costs* [25], and they could be the time, energy, and money that the poor need to maintain the connection to digital devices. One example of such costs could be ensuring that bills are paid on time to ensure continuous internet or phone connectivity, which previous research has documented as the key impediment to successful adoption of electronic health (eHealth) interventions [26]. In addition, wearable gadgets and health apps often work best on the latest operating systems, and if the poor are not able to spend more money to get the latest gadgets to obtain the latest updates, they would be systematically left out. Without consideration for these costs, studies have shown that even with the provision of technology and internet access, the underprivileged groups still faced significant barriers in taking advantage of big data and new technologies that would significantly improve their health [27] if they are unable to pay for continuous access. Studies have documented that when the underprivileged groups were unable to pay their phone bills, it had severe ramifications, as frequent changes in phone numbers would result in disrupted care, leading to missed appointments and important paperwork (eg, insurance claims) deadlines [25].

To alleviate these latent costs, researchers should be mindful to factor in an additional budget to reduce the connection maintenance costs borne by the underprivileged groups, such as covering their cell phone bills for health app interventions. For instance, in a study examining health information seeking habits among the underprivileged groups, the researchers conducted a randomized controlled trial to examine if provision of home computers, broadband internet access, training in computer use, and a Web portal designed for low-literacy populations would significantly improve internet use [13]. The results showed that participants in the intervention group (ie, those who received computers, internet access, computer training, and a Web portal) were more likely to use the internet compared with the control group. This demonstrates that when researchers are mindful in addressing hidden costs (eg, bills for internet connection) that participants need to bear to be a part of big data research projects, it would significantly reduce structural barriers that prevent them from fully engaging with the research.

## Organizational-Level Context: Forging Strategic Data Alliances

Next, one of the key strategies for health organizations—regardless if they are well resourced or not—is to take active steps to forge strategic data alliances with other organizations that leverage their comparative advantage and circumvent their own organizational constraints. For instance, although large health institutions may have the resources to implement big data solutions and research, they may not be as effective as community health centers in reaching out to the poor [28]. Small health organizations (eg, community health centers), on the contrary, may not have the necessary training or infrastructure to use big data. A recent study examining rural public health system leaders’ data needs in Alaska, Idaho, Oregon, and Washington found that they were ill equipped in data management and had limited experience with data analysis [29]. However, they would be valuable to large health organizations because of their access, experience, expertise, and relationship of trust established with the underprivileged groups [30]. Although this is easier said than done, there are a few practical ways to do this:

- Create Communities of Practice (CoP) where health organizations could come together periodically (eg, annually) to share best practices of big data use in addressing health disparities, their challenges, and identify strategies to engage the underprivileged groups.
- Through the CoP, build a mentorship culture where personnel from organizations that are further along in their big data journey could mentor staff from health organizations that are getting started using big data for health disparities.

One potential example of a CoP is the recent launch of a US $100 million initiative by the Rockefeller Foundation and other global health partners that aim to specifically empower frontline community health workers with the most affordable and latest innovations in data science for improving health [31]. Part of the initiative would entail creating a knowledge and data sharing network where partnering countries could tap into a global team of data science experts committed to sharing of technical expertise and resources in the context of improving community health.

## Individual-Level Context: Building Trust With the Underprivileged Groups

Recognizing that issues of privacy violation, loss of confidentiality, and data abuse [32] are some of the reasons at the individual level for mistrust and cynicism in how big data are used in the health care system, it is crucial that health organizations prioritize establishing trust with the underprivileged groups. To do so, health organizations should strengthen communication efforts such that literacy support should be provided for any informatics intervention [9], and the tangible benefits to participants and their communities should be made clear without jargon. Previous research that used eHealth interventions in community settings with people from underprivileged groups found that in-person presentations and personal contact with community members and organizations were the most effective in recruitment and participation [26]. In other words, the design of health big data research should incorporate *people-powered data collaboratives*, where end users or beneficiaries of health big data should be treated as stakeholders and brought to the table from the get-go to give them a stake in deciding how and when their data could be used on their own terms [33]. Eliciting a higher degree of participation and engagement from the underprivileged groups would strengthen relationships and cultivate a group identity and possibly a sense of belonging [34], thereby enhancing greater trust.

An example of this is the *All of Us* research program led by the National Institutes of Health in the United States, which aims to gather lifestyle, environmental, and biological data—Electronic Health Records (EHRs), blood samples, and information from wearables and surveys—from 1 million or more people from diverse groups in the United States to improve biomedical research to advance health [35]. To improve trust with participants, researchers provided participants access to their own data and the results of any laboratory tests they undertook [36]. In addition, the researchers sought participants’ feedback (in addition to experts) when drafting guidelines and frameworks on how the data could be better communicated with others.

## Data Context: Prioritize Science Over Data in the Use of Data Science

Although understanding societal-, organizational-, and individual-level contexts would address data absenteeism, what can researchers do to avoid falling into the trap of data chauvinism? Ultimately, researchers within health organizations should prioritize scientific rigor in their use of data. There are 3 practical ways to do so. First, researchers should balance the *a priori* rigor of scientific inquiry with a data-driven paradigm and understand the context in which one would perform better than the other. The a priori scientific inquiry is the traditional scientific hypothesis testing approach where researchers first develop a set of research questions and hypotheses and set out to mine data to verify their assumptions. The data-driven paradigm draws much from existing machine learning approaches that seek to mathematically detect patterns in the data through the process of data wrangling, as well as refining algorithms from training datasets so that it could effectively predict outcomes [23]. Although there is nothing inherently wrong with this data-centric method, the danger of the current big data hype is the move toward a puritanical pursuit of being data driven at the expense of crowding out subject or domain experts or common sense. In the case of GFT, perhaps by taking a step back and asking the fundamental question of how reliable search queries were in serving as leading indicators of realities, it might attenuate the way the Google engineers thought about designing the algorithms and thus avoid the serious inflation of results.

Second, part of emphasizing the rigor of science is to consider data from multiple sources. After all, big data are not only about the volume but also the variety of sources. In the GFT example, one of the pitfalls was implicit algorithmic snobbery, where data and algorithms from Google were treated as superior compared with lagged data from the CDC. If, in the first place, the Google algorithms were dynamically recalibrated with CDC data (despite their limitations), it could have avoided the problem of overestimation [22].

Finally, health organizations should take steps to implement a data quality assessment framework, where researchers could evaluate their data in the context of the big questions on health disparities they are addressing. In this data quality assessment framework, researchers should go beyond addressing questions on why or what variables have missing values and aim to answer how effective the data are in helping researchers address the root causes of health disparities. For instance, although the application of machine learning and artificial intelligence algorithms on EHRs may tell us which patients from underprivileged groups are more likely to get readmitted to hospitals for the same problem, the data would not empower health care providers to assess how best to alleviate the conditions to prevent readmissions. Thus, a rigorous quality assessment of big data in health disparities should guide researchers from simply asking, “what can these data tell us” to “how can these data points reduce disparities and what additional data would be required?”

## Conclusions

The potential to actualize the promises of big data in bridging health disparities to some extent is contingent on health organizations’ efforts to address data absenteeism and data chauvinism. Although there are no easy solutions, it is crucial for health organizations to be keenly aware of both problems and develop a firm contextual understanding as well as coordinate strategies at the societal, organizational, individual, and data levels. Certainly, we agree that in the era of big data, taking small steps is crucial for success [37]; it also requires fundamental paradigm and attitudinal shifts within health organizations. Ironically, successful big data use in health disparities would require health organizations to look beyond data itself and to be intentionally inclusive so that no one is left behind so that the underprivileged could become the beneficiaries in the data revolution.

## Abbreviations

CDC: Centers for Disease Control and Prevention
CoP: Communities of Practice
eHealth: electronic health
EHR: electronic health record
GFT: Google Flu Trend

## References

[1] Hay SI, George DB, Moyes CL, Brownstein JS (2013). Big data opportunities for global infectious disease surveillance. PLoS Med.

[2] Kuehn BM (2015). Twitter streams fuel big data approaches to health forecasting. J Am Med Assoc.

[3] Farrington C (2016). Big data meets human health: Internet searches and fitness trackers are poised to play a role in the future of health care. Science.

[4] Perzynski AT, Roach MJ, Shick S, Callahan B, Gunzler D, Cebul R, Kaelber DC, Huml A, Thornton JD, Einstadter D (2017). Patient portals and broadband internet inequality. J Am Med Inform Assoc.

[5] Wang Y, Kung L, Byrd TA (2018). Big data analytics: Understanding its capabilities and potential benefits for healthcare organizations. Technol Forecast Soc Change.

[6] Chang WL, Grady N, NIST Big Data Public Working Group (2015). NIST: National Institute of Standards and Technology.

[7] Onukwugha E, Duru OK, Peprah E (2017). Foreword: big data and its application in health disparities research. Ethn Dis.

[8] Carney TJ, Kong AY (2017). Leveraging health informatics to foster a smart systems response to health disparities and health equity challenges. J Biomed Inform.

[9] Veinot T, Mitchell H, Ancker J (2018). Good intentions are not enough: how informatics interventions can worsen inequality. J Am Med Inform Assoc.

[10] Kontos EZ, Emmons KM, Puleo E, Viswanath K (2010). Communication inequalities and public health implications of adult social networking site use in the United States. J Health Commun.

[11] Breen N, Jackson JS, Wood F, Wong DW, Zhang X (2019). Translational health disparities research in a data-rich world. Am J Public Health.

[12] Ghassemi M, Naumann T, Schulam P, Beam AL, Chen IY, Ranganath R (2019). Practical guidance on artificial intelligence for health-care data. Lancet Digit Heal.

[13] Viswanath K, McCloud R, Minsky S, Puleo E, Kontos E, Bigman-Galimore C, Rudd R, Emmons KM (2013). Internet use, browsing, and the urban poor: implications for cancer control. J Natl Cancer Inst Monogr.

[14] Cohn EG, Hamilton N, Larson EL, Williams JK (2017). Self-reported race and ethnicity of US biobank participants compared to the US Census. J Community Genet.

[15] Ng JH, Ye F, Ward LM, Haffer SC, Scholle SH (2017). Data on race, ethnicity, and language largely incomplete for managed care plan members. Health Aff (Millwood).

[16] Bol N, Helberger N, Weert JC (2018). Differences in mobile health app use: a source of new digital inequalities?. Inf Soc.

[17] Viswanath K, Kreuter MW (2007). Health disparities, communication inequalities, and eHealth. Am J Prev Med.

[18] Hargittai E (2018). Potential biases in big data: Omitted voices on social media. Soc Sci Comput Rev.

[19] Sarkar U, Karter AJ, Liu JY, Adler NE, Nguyen R, López A, Schillinger D (2011). Social disparities in internet patient portal use in diabetes: evidence that the digital divide extends beyond access. J Am Med Inform Assoc.

[20] Moran MB, Frank LB, Zhao N, Gonzalez C, Thainiyom P, Murphy ST, Ball-Rokeach SJ (2016). An argument for ecological research and intervention in health communication. J Health Commun.

[21] Confessore N (2018). The New York Times.

[22] Lazer D, Kennedy R, King G, Vespignani A (2014). Big data. The parable of Google Flu: traps in big data analysis. Science.

[23] Kreatsoulas C, Subramanian S (2018). Machine learning in social epidemiology: learning from experience. SSM Popul Health.

[24] Featherson L (2018). The New York Times.

[25] Gonzales AL, Ems L, Suri VR (2016). Cell phone disconnection disrupts access to healthcare and health resources: a technology maintenance perspective. New Media Soc.

[26] Nagler RH, Ramanadhan S, Minsky S, Viswanath K (2013). Recruitment and retention for community-based eHealth interventions with populations of low socioeconomic position: strategies and challenges. J Commun.

[27] McCloud RF, Okechukwu CA, Sorensen G, Viswanath K (2016). Beyond access: barriers to internet health information seeking among the urban poor. J Am Med Inform Assoc.

[28] Boutin-Foster C, Scott E, Melendez J, Rodriguez A, Ramos R, Kanna B, Michelen W (2013). Ethical considerations for conducting health disparities research in community health centers: a social-ecological perspective. Am J Public Health.

[29] Bekemeier B, Park S, Backonja U, Ornelas I, Turner AM (2019). Data, capacity-building, and training needs to address rural health inequities in the Northwest United States: a qualitative study. J Am Med Inform Assoc.

[30] Stephens KK, Rimal RN, Flora JA (2004). Expanding the reach of health campaigns: community organizations as meta-channels for the dissemination of health information. J Health Commun.

[31] The Rockefeller Foundation (2019). The Rockefeller Foundation.

[32] Zhang X, Pérez-Stable EJ, Bourne PE, Peprah E, Duru OK, Breen N, Berrigan D, Wood F, Jackson JS, Wong DW, Denny J (2017). Big data science: opportunities and challenges to address minority health and health disparities in the 21st century. Ethn Dis.

[33] Evans B, Krumholz H (2019). People-powered data collaboratives: fueling data science with the health-related experiences of individuals. J Am Med Inform Assoc.

[34] Ramanadhan S, Mendez SR, Rao M, Viswanath K (2013). Social media use by community-based organizations conducting health promotion: a content analysis. BMC Public Health.

[35] (2019). National Institutes of Health.

[36] Denny JC, Rutter JL, Goldstein DB, Philippakis A, Smoller JW, Jenkins G, Dishman E, All of Us Research Program Investigators (2019). The 'All of Us' Research Program. N Engl J Med.

[37] Heller N, Seltzer JH (2017). Commentary: in the rising era of big data, small steps are key. Ethn Dis.

